# Quality of life, satisfaction and outcomes after ministernotomy versus full sternotomy isolated aortic valve replacement (QUALITY-AVR): study protocol for a randomised controlled trial

**DOI:** 10.1186/s13063-018-2486-x

**Published:** 2018-02-17

**Authors:** Emiliano A. Rodríguez-Caulo, Ana Guijarro-Contreras, Juan Otero-Forero, María José Mataró, Gemma Sánchez-Espín, Arantza Guzón, Carlos Porras, Miguel Such, Antonio Ordóñez, José María Melero-Tejedor, Manuel Jiménez-Navarro

**Affiliations:** 1 UGC Área del Corazón. Servicio de Cirugía Cardiovascular y Cardiología. Hospital Universitario Virgen de la Victoria de Málaga, Spain. Fundación Pública Andaluza para la Investigación de Málaga en Biomedicina y Salud (FIMABIS). Universidad de Málaga. Spain. CIBERCV Enfermedades Cardiovasculares, Instituto de Salud Carlos III, Madrid, Spain; 2 UGC Área del Corazón, Servicio de Cirugía Cardiovascular, Hospital Universitario Virgen del Rocío de Sevilla, Red de Investigación Cardiovascular (RIC), Instituto de Salud Carlos III, Madrid, Spain

**Keywords:** Aortic stenosis, Ministernotomy, Aortic valve replacement, Clinical trials, Quality of life, Satisfaction

## Abstract

**Background:**

During the last decade, the use of ministernotomy in cardiac surgery has increased. Quality of life and patient satisfaction after ministernotomy have never been compared to conventional full sternotomy in randomised trials. The aim of the study is to determine if this minimally invasive approach improves quality of life, satisfaction and clinical morbimortality outcomes.

**Methods/design:**

The QUALITY-AVR trial is a single-blind, single-centre, independent, and pragmatic randomised clinical trial comparing ministernotomy (“J” shaped upper hemisternotomy toward right 4th intercostal space) to full sternotomy in patients with isolated severe aortic stenosis scheduled for elective aortic valve replacement. One hundred patients will be randomised in a 1:1 computational fashion. Sample size was determined for the primary end point with alpha error of 0.05 and with power of 90% in detecting differences between intervention groups of ≥ 0.10 points in change from baseline quality of life Questionnaire EuroQOL-index (EQ-5D-5 L®), measured at 1, 6 or 12 months. Secondary endpoints are: the differences in change from other baseline EQ-5D-5 L® utilities (visual analogue scale, Health Index and Severity Index), cardiac surgery specific satisfaction questionnaire (SATISCORE®), a combined safety endpoint of four major adverse complications at 1 month (all-cause mortality, acute myocardial infarction, neurologic events and acute renal failure), bleeding through drains within the first 24 h, intubation time, postoperative hospital and intensive care unit length of stay, transfusion needs during the first 72 h and 1-year survival rates. Clinical follow up is scheduled at baseline, 1, 6, and 12 months after randomization. All clinical outcomes are recorded following the *Valve Academic Research Consortium 2 criteria.*

**Discussion:**

The QUALITY-AVR trial aims to test the hypothesis that ministernotomy improves quality of life, satisfaction and clinical outcomes in patients referred for isolated aortic valve replacement. Statistically significant differences favouring ministernotomy could modify the surgical “gold standard” for aortic stenosis surgery, and subsequently the need to change the control group in transcatheter aortic valve implantation trials. Recruitment started on 18 March 2016. In November 2017, 75 patients were enrolled.

**Trial registration:**

ClinicalTrials.gov, NCT02726087. Registered on 13 March 2016.

**Electronic supplementary material:**

The online version of this article (10.1186/s13063-018-2486-x) contains supplementary material, which is available to authorized users.

## Background

The progressive ageing of the population has caused an increase in aortic valve procedures due to degenerative diseases [1], while mortality has decreased thanks to improvements in surgical techniques [2]. The current “gold standard” is conventional surgery with a full median sternotomy (FS) to replace the aortic valve. In the last decade, to minimise invasiveness and improve outcomes, there has been an increase in the use of smaller incisions such as the ministernotomy (MS) and new technologies (transcatheter aortic valve implantation (*TAVI*), or sutureless valves).

**Fig. 1:**
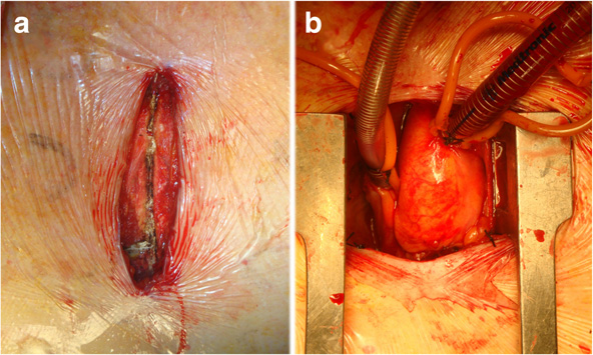
Ministernotomy extended in a J-shape into the right fourth intercostal space. **a** Before sawing. **b** Surgical view after sawing, retraction and cannulation

Minimally invasive aortic valve replacement (AVR) surgery was first described in 1993 [3] and popularised between 1996 and 1997 [4] as an alternative to FS for patients with isolated aortic valve or ascending aorta pathologic conditions. Various techniques have been described, although currently the most frequently used is MS (partial upper hemisternotomy extended in a J-shape into the right fourth intercostal space, Fig. 1) [5]. In 2008, the American Heart Association defined minimally invasive surgery as “a small chest wall incision that does not include the conventional FS” [6].

To date, few clinical trials have been conducted that compare AVR surgery using MS versus FS [7–11]. No significant differences have ever been found, due to inadequate design, lack of statistical power or too small a sample size for the primary endpoint of mortality, even though there have been significant differences in morbidity with MS (lower rates of pain, transfusions, bleeding, mechanical ventilation time, stay in intensive care and hospital, etc.) To detect differences in mortality with sufficient power, over 1100 patients would be needed for each branch of the study. This circumstance caused a spate of retrospective studies analysing propensity scores, and meta-analysis [12–14], which confirmed a reduction in postoperative morbidity, and demonstrated a decrease in early [13–15], and even in late, mortality [13]. But there is an evident bias inherent to retrospective studies. This increased the use of MS worldwide, above all as a result of the spread of TAVIs.

There have been no prospective studies to measure patient quality of life (QOL) and compare the techniques of MS versus FS for AVR. There has only been one retrospective study, with results that are not statistically significant [16]. Two clinical trials have compared TAVI versus FS (the PARTNER [17] and the Core Valve PIVOTAL trials [18]) but they did not analyse MS. QOL has always been relevant to the patient; however, clinicians have not given it enough relevant focus, and it is usually and statistically significant in the first month in favour of TAVI [17, 18], with all data coming from secondary analysis, not primary endpoints.

Patient-reported QOL outcomes were selected to be the primary endpoint because if similar recovery time and QOL to TAVI is demonstrated, MS provides the capacity of implantation of more durable valves; therefore, it would be used in patients at low and intermediate risk, given the unknown long-term durability of TAVI valves after 5 years. To date, however, there has been no specifically designed QOL study. For all of these reasons, we designed this clinical trial to compare the QOL after MS versus FS. Satisfaction with the surgery and morbimortality outcomes will also be measured.

## Methods/design

**Fig. 2:**
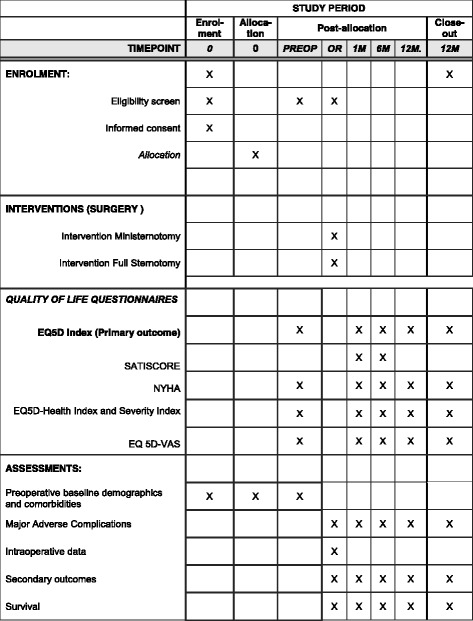
Standard protocol items: recommendation for interventional trials (SPIRIT) figure of participant timeline. EQ5D, Euroqol 5 dimensions Quality of life Questionnaire; M, month; NYHA, New York heart Association; OR, surgery in the operating room; PREOP, preoperative; VAS, visual analogue scale

This randomised clinical trial follows the standard protocol items: recommendation for interventional trials (SPIRIT) guidelines (see Additional file 1). The study schedule of enrolment, interventions, and assessments is presented in Fig. 2.

### Study design and endpoints

This is a pragmatic [19], independent (not industry supported), single-blind (patient), single centre, randomised clinical trial that compares two treatment groups: patients undergoing AVR with FS or with MS. The study was approved by our Institution’s Ethics Committee for Research (Institutional Review Board), and was registered at ClinicalTrials.gov **(NCT02726087**) before the first patient was randomised on 18 March. At January 2018, current protocol version is 1.4 after minor changes were performed.

### Quality of life and satisfaction questionnaires

The outcome of heart surgery may be evaluated by means of “hard” outcomes, such as mortality, survival, morbidity and heart function, or at different points in time, but the impact of the operation is not merely limited to these biological aspects. An adequate assessment should cover other areas, such as psychological and social aspects, which requires the use of psychometric tools, such as questionnaires. Within these areas, one of the most important is assessment of patient QOL, whereby questionnaires are used to explore physical, psychological and emotional aspects.

There are a multitude of questionnaires including the Short Form 36-item Health Survey [20], the Minnesota Living with Heart Failure (MLWHF) [21], the Short Form 12 [22], the Kansas City Cardiomyopathy Questionnaire (KCCQ) [23], and the EuroQoL 5 Dimensions 5 Levels (EQ-5D-5 L®) [24]. We chose the latter as it had been tested in the Spanish population in the Spanish National Health Survey 2011–2012. It was validated for cardiovascular pathologies and it can measure a health unit, the quality-adjusted life year (QALY).

**The EQ-5D-5 L® questionnaire** presents various utilities to measure QOL. The main ones are a descriptive system that can be used to calculate an EQ-index and the visual analogue scale (**VAS**); other utilities may also be calculated such as the severity index or the health index. This questionnaire estimates the state of health in 5 dimensions (mobility, self-care, usual activities, pain/discomfort and anxiety/depression), with five possible responses depending on extent (5 levels: no problems, slight problems, moderate problems, severe problems, unable to /extreme problems). The **EQ-5D-5 L® index** is calculated via the application, the EQ-5D-5 L® Crosswalk Index Value Calculator, using a logistic regression model. Thus, EQ-5D-5 L® (5 dimensions on 5 levels) distinguishes 3125 states of health. The index takes values between 1 (state of health 11111) and − 0.654 (state of health 55555) in the Spanish population.

The subjective state of health is estimated using the VAS of 0 to 100, where 0 is the worst imaginable health and 100 is the best imaginable health.

The **Severity Index (SI)** is obtained by adding the digits that correspond to the levels of the 5 dimensions in each state of health, subtracting 5 and multiplying by 5, which produces a new index (0–100), where 0 indicates a total absence of health problems and 100 is the highest degree of severity. Subtracting the SI from 100 will give the **Health Index (HI).**

As soon as the patient has signed the consent form, the baseline preoperative questionnaires will be completed, which will be repeated at 1, 6 and 12-month follow up. All questionnaires can be completed in person or by telephone interview.

**The SATISCORE® questionnaire** [25] assesses patient satisfaction after undergoing heart surgery. It analyses six dimensions of satisfaction with the surgery: vitality, sociability, mood, sexuality, self-perception and rest. It consists of twenty statements (Table 1) with 6 possible responses on a Likert scale: 0, no answer; 1, very unsatisfied; 2, unsatisfied; 3, don’t know; 4, satisfied; and 5, very satisfied. Scores range from 0 to 100 and it is valid specifically to evaluate the satisfaction of patients who have undergone heart surgery. It is completed at 1 month and 6 months post-surgery.

**Table 1:** Items included in the SATISCORE® questionnaire

Feeling of illness	
Pain	
Feeling of fatigue	
Feeling when you get up in the morning	
Exercise you have been indicated	
Pharmacological treatment	
Feeling about the operation	
Feeling of tiredness	
Your state of health	
Perception of future health	
Attitude to your illness	
The life you lead today	
Feeling about the operation	
Family relationships	
Friendships	
Medical assistance received in the hospital	
Willingness to repeat the operation	
Future plans	
Sex life	
Precaution in sexual relations due to the operation	

### Selection of patients, randomisation and follow up

The study will recruit patients over the age of 18 years, who have severe aortic stenosis or double aortic lesion with predominant stenosis, who are symptomatic according to current guidelines [26] and also meet the inclusion and exclusion criteria (Table 2). All patients will receive oral and written information from a staff surgeon about the study and then as an informed patient sign a consent form before their inclusion and randomisation in the study. These data were recorded in the hospital computerized healthcare database.

**Table 2:** Inclusion and exclusion criteria

Inclusion criteria
• Severe aortic stenosis (calcified aortic valve, aortic valve area < 1 cm^2^ or body surface area index < 0.6 cm^2^, mean transvalvular gradient > 40 mmHg or peak systolic velocity > 4 m/s) or double aortic lesion with predominance of stenosis
• Sympotomatic (dyspnea NYHA score ≥ 2, angina or syncope)
• Age ≥ 18 years
• Capacity to give informed consent
Exclusion criteria
• Moderately depressed ejection fraction (< 40%)
• Prior heart surgery (redo operation)
• Emergent surgery (within the first 24 h of admission)
• Infectious endocarditis
• Chronic obstructive pulmonary disease greater than moderate (forced expiratory volume at 1 s (FEV1) predicted < 60% measured by spirometry)
• Need for concomitant surgery except Morrow myectomy preoperative or intraoperative

Randomisation is performed using a randomisation computer programme, in blocks of four patients in a ratio of 1:1. Allocation concealment is achieved by an administrative officer who maintains custody of the randomisation sequence (sequentially numbered), and communicates to medical staff the treatment assigned. This is performed after the consent form has been signed and the surgery scheduled. The patient is blinded with respect to the treatment received. All wounds will be covered up to this moment with standardised wound dressings. There is great difficulty in blinding surgical procedures for a long time, because incisions and scars may differ between groups [27]. That is why the size of the wounds in both arms will be intended to be as small as possible, about 10 cm in the ministernotomy and 13–15 cm in conventional surgery. A standardized hospital discharge report will be used, without breaking the blinding unless it is strictly necessary due to medical needs, up to the final follow-up assessment at 1 year post-surgery. With these measures we will try to reduce the risk of bias, which will always be present in this type of study involving surgical procedures (for example with a surgeon who is not blinded to procedure). The outcome assessor is the Hospital Clinical Trials Data Monitoring Unit, independent from the Sponsor, which reviews and confirms all the outcomes and endpoint values every 3 months. Both groups of patients will receive clinical follow up and complete the EQ-5D-5 L® quality of life questionnaire at 1, 6 and 12 months, and the SATISCORE® at 1 and 6 months.

Any changes to the research protocol will be reviewed by the Institutional Review Board (IRB). Amendments will be made to the trial registry as necessary.

### Study endpoints

The primary endpoint measure is to detect differences between MS (active treatment) and FS (control) greater than or equal to 0.10-point change from the baseline questionnaire EQ-5D-5 L® Index, at 1, 6 or 12 months after the surgery.

The secondary endpoint measures are:

- Differences between intervention groups greater than or equal to 10 point-change from the baseline questionnaire EQ-5D-5 L® utilities such as VAS, SI and HI at 1, 6 or 12 months post-surgery.
- Early postoperative combined endpoint of four major adverse complications (MAC) at 1 month (safety endpoint), including all-cause mortality, acute myocardial infarction, stroke or transient ischaemic accident, and classification of acute renal failure by Acute Kidney Injury Network (AKIN) greater than or equal to 2. Follow up at 1 year post-surgery.
- Severe nosocomial infections (pneumonia, early endocarditis, mediastinitis, sepsis).
- Need for rehospitalisation.
- Differences between interventions groups greater than or equal to 10 points change from the baseline SATISCORE® questionnaire at 1 and 6 months post-surgery.
- Postoperative hospital length of stay.
- Postoperative intensive care unit length of stay.
- Times for ischaemia and cardiopulmonary bypass (CPB).
- Mechanical ventilator support after surgery (intubation times).
- Bleeding in the first 24 h after surgery and transfusion requirements in the first 72 h.
- New York Heart Association (NYHA) functional class at 1 to 6–12 months.
- Survival at 12 months.

### Sample size calculation

A review of previous work [28, 29] shows that the standard deviations of the EQ-5D-5 L® index in cardiovascular disease varies between 0.10 and 0.22, while VAS score varies between 8 and 21 depending on the severity. Likewise, in the only (multicentre) study [17] that determines quality of life in aortic stenosis, the standard deviation for high-risk patients scheduled for AVR was 0.17 in patients randomised to FS versus TAVI. For these reasons, on summary of all previous work, we used a standard deviation of 0.15 for the EQ-5D-5 L® index to calculate the sample size.

In QOL studies, it is not only important to ascertain whether there are significant differences, but also to know if these differences are clinically relevant. This is why the concept of the **minimal important difference (MID)** was created, which in previous research among patients with cancer was 0.08 points on the EQ-5D index and 7–11 points on the VAS [29], and in patients with stroke it was 0.10 points on the EQ-index (95% CI 0.08–0.12) [28]. This difference was also 0.06 points (95% CI 0.02–0.10) on the EQ index during the first month in the PARTNER 1 study (transfemoral TAVI versus FS) [17], and 0.12 (95% CI 0.08–0.16) in the CoreValve PIVOTAL trial study [18]. As there was no existing specific calculation of MID for cardiology patients with severe aortic stenosis, we arbitrarily established the interval of 0.10 points on the EQ-5D index (the mean value from previous studies) as the clinically relevant difference to detect. Thus, to detect an MID of at least 0.10 points in the QOL scale EQ-5D-5 L® index, with alpha error of 0.05, beta error of 0.1 and power of 90% for two-tailed contrast of two independent means (SD 0.15), two groups of 48 patients are necessary to provide a minimum of 96 patients. In view of possible losses to follow up, 100 patients will be randomised. If patients drop out or are excluded during the study, additional patients will be recruited until a minimum of 96 patients is achieved, and inclusion of an additional patient will be considered again for each patient not completing the 1-year follow-up assessment.

### Description of the surgical procedures

#### Conventional full median sternotomy

The patient is positioned supine (dorsal decubitus). The skin is incised from the suprasternal notch to the xiphoid process. Conventional median sternotomy is performed from the manubrium to the xiphoid. The ascending aorta and right atrium are cannulated centrally to initiate CPB. A vent cannula for the left-sided cavities is placed in the right superior pulmonary vein, followed by aortic cross-clamping. Intermittent cold antegrade blood cardioplegia via the root or coronary ostia every 20 min, transverse aortotomy, valve extraction and decalcification of the annulus are performed. AVR is performed using Ti-Cron 2/0® polyester suture stitches supported by Teflon in an aortic ring. The aortotomy is closed using polypropylene monofilament 4/0, followed by aortic unclamping, decannulation and placement of a transitional pacemaker placement. Placement of two sub -xiphoid Blake type drains. Sternal closure with stainless steel wires. The skin incision is closed with a subcutaneous double layer and staples or intradermic suture.

#### Ministernotomy

The patient is positioned supine (dorsal decubitus) and defibrillator external paddles are positioned. An 8 to 10-cm opening is made in the skin starting at the sternal angle. Ministernotomy is defined as a partial upper hemisternotomy extended into a J-shape into the right fourth intercostal space irrespective of the skin incision (usually 8–12 cm in length, Fig. 1). A 21-mm silicon Blake-type drain is inserted sub-xiphoid. CO2 is administered via the sub-xiphoid drain placed before the CPB was established. The rest of the procedure is similar to that used for FS.

### Independent variables in the study. Complications

Data on preoperative baseline demographic variables will be recorded in both intervention groups. Most of the definitions of postoperative complications are to be found in the consensus document, *Valve Academic Research Consortium 2* (VARC2) [30] that arose as a consequence of the generalisation of TAVI, and the need for consistency of definitions and improved comparability of articles. The AKIN classification was used in the VARC 2 criteria according to the European Society of Cardiology (ESC)/European Association for Cardio-Thoracic Surgery (EACTS) recommendations, and it is the only way to compare new data with previous reports/trials of TAVI [30].

### Statistical analysis

Statistical analysis will be performed using IBM SPSS 22.0® for Windows®. First, a descriptive analysis of the study variables will be conducted; the values of quantitative variables will be expressed as mean ± standard deviation (SD) or median (interquartile range) depending on whether distribution of the variable is symmetrical and normal. Qualitative variables will be presented in absolute frequencies and relative frequencies. To analyse the differences between continuous quantitative variables (values of the EQ-5D-5 L® and SATISCORE questionnaires) in the two independent groups (MS vs. FS), Student’s *t* test for two independent samples will be applied in cases where the normality of distribution of the variable data in each of the groups can be accepted, which will be checked using the Kolmogorov-Smirnov test, then histograms and Q-Q plots. In cases where normality cannot be accepted, the corresponding non-parametric Wilcoxon test will be applied. In cases where the normality condition can be accepted, Student’s *t* test for two-tailed samples will be applied to analyse differences between continuous quantitative variables in two associated groups (QOL data) before and after surgery.

We will apply analysis of variance (ANOVA) for repeated measures to analyse the variability, with repeated measurements over time, of certain continuous quantitative determinations of the questionnaires used. The normality of the remainder will be checked using the Shapiro-Wilk test. In the case of statistically significant differences in the contrasted variables measured at different times, the different levels of time will be checked, adjusting the outcomes using the Bonferroni correction.

To analyse if there are differences between qualitative variables, 2 × 2 tables will be prepared and analysed using the Chi-squared (*X*2) statistical test. This analysis will be performed to evaluate the differences in complications and major adverse events of dichotomous variables. The relative risk ratio and the corresponding confidence intervals will be calculated to 95% for the MS versus FS technique as a combined safety endpoint of MAC complications.

Multivariate regression analysis techniques will be applied to control possible bias in effect caused by the patient’s initial situation (comorbidities). Pearson’s correlation test will be performed on EQ-5D-5 L® and SATISCORE® data. Survival analysis will be conducted using Mantel Cox log-rank analysis with the corresponding Kaplan-Meier survival chart.

The “intention to treat” population is defined to include all randomised subjects. This population will be used for endpoint analyses. The as-treated population (“per protocol”) is defined to include all subjects undergoing the index procedure. This population will be used for the analysis of adverse events.

Differences between MS and FS group scores at each follow-up time point will be estimated with longitudinal random-effect growth curve models, which will be fit to the repeated measurements for each health status outcome. These longitudinal analyses will use all available quality-of-life data: including data from patients who will subsequently die, withdraw, or will be lost to follow up. They will also accommodate missing data under the missing at random assumption. Variables that will be included in the models are: treatment assignment, pre-specified patient characteristics (age, sex, EuroScore), follow-up time and interactions between treatment and time. These models will be used to calculate the mean between-group differences in the EQ-5D index score and the individual subscales at each follow-up time point, and the associated 95% CIs and *P* values.

To examine the potential impact of missing data, which, given the illness severity of the trial population, would most likely not be missing at random, we will repeat the growth curve analyses after imputing the missing scores among the surviving patients, as the lowest reported score among respondents within each treatment group for each respective time point. We also will examine the magnitude of potential survivor bias by comparing the mean baseline scores between treatment groups for the subgroups of patients with available QOL data at each successive follow-up time point.

### Dissemination

Trial results will be posted on ClinicalTrials.gov, published in peer-reviewed journals, and presented at national conferences. Data-sharing policy will be under request to the Sponsor and publicly explained and recorded at ClinicalTrials.gov.

### Limitations of the study

Among this study’s limitations, there could be some confusion bias introduced by variables, which will be controlled in the statistical analysis using multivariate linear regression analysis. The absence of masking the surgeon may introduce an unmeasurable bias into the study. The sample size is not designed to detect differences in mortality, although a combined safety endpoint such as this one may help to find differences between groups. The use of general QOL questionnaires such as EQ-5D-5 L means that the outcome does not depend exclusively on the surgical technique employed, but also on other diseases and/or complaints that the patient has previously or that may be acquired in the future, independently of the heart condition. The size of the sample could also be insufficient if the standard deviations are shown to be higher than those presented in previous research.

## Summary and trial status

The QUALITY-AVR trial (NCT02726087) aims to test the hypothesis that MS improves QOL, satisfaction and clinical outcomes in patients referred for isolated AVR. The design of this study is unique to date, as there is no randomised clinical trial to have compared these outcomes. We hope that the results of the study can influence the future of AVR surgery and encourage the adoption of a new surgical “gold standard” for MS, with improved QOL and outcomes, in the short and long term. If this proves to be the case, clinical trials comparing TAVI to surgery should include MS to evaluate outcomes in low and intermediate risk patients as the “control” group. Recruitment began in March 2016 (75 patients currently recruited at November 2017) and it is expected to end in May 2018. All outcomes will be published within 2 years in accordance with Consolidated Standards on Reporting Trials (CONSORT) recommendations.

## Additional file

Additional file 1: SPIRIT 2013 checklist: Recommended items to address in a clinical trial protocol and related documents. (DOCX 52 kb)

## Abbreviations

AKIN: Acute Kidney Injury Network
AVR: Aortic valve replacement
CBP: Cardiopulmonary bypass
EQ-5D-5 L: EuroQOL Quality of life Questionnaire 5 dimensions 5 Levels
FS: Full sternotomy (conventional surgery)
HI: Health Index
MAC: Major adverse complications
MS: Mini sternotomy
QOL: Quality of life
SI: Severity Index
*TAVI*: Transcatheter aortic valve implantation
VAS: Visual analogue scale
